# Clinical, pathological and prognostic characteristics of gastroenteropancreatic neuroendocrine neoplasms in China: a retrospective study

**DOI:** 10.1186/1472-6823-14-54

**Published:** 2014-07-08

**Authors:** Xianbin Zhang, Li Ma, Haidong Bao, Jing Zhang, Zhongyu Wang, Peng Gong

**Affiliations:** 1 Department of Hepatobiliary Surgery, the First Affiliated Hospital of Dalian Medical University, Zhongshan Road No. 222, Dalian 116011, Liaoning Province, China; 2 Department of Epidemiology, Dalian Medical University, Dalian 116044, Liaoning Province, China

**Keywords:** Neuroendocrine neoplasms, Carcinoma, Epidemiology, China

## Abstract

**Background:**

Gastroenteropancreatic neuroendocrine neoplasms (GEP-NENs) are rare neuroendocrine tumors, and lack of data in Asian populations especially in China. The aim of this retrospective study was to assess the clinical, pathological and prognostic characteristics of GEP-NENs in China.

**Methods:**

We collected clinical and pathological data of 168 patients diagnosed with GEP-NENs and treated at the First and Second Affiliated Hospitals of Dalian Medical University between January 2003 and December 2012. Kaplan-Meier method and log rank analysis was used to analyze the prognostic significance of clinical and pathological characteristics.

**Results:**

Mean age was 51.83 ± 14.03 and the male-to-female ratio was 1.5:1. Primary sites were the rectum (58.93%), pancreas (13.69%), stomach (9.52%), duodenum (5.36%), colon (4.76%), appendix (4.76%), ileum (2.38%) and jejunum (0.60%). Most patients (95.83%) presented non-functional tumors with non-specific symptoms such as abdominal or back pain (29.17%) and gastrointestinal bleeding (25.60%). Based on the 2010 World Health Organization (WHO) classification, patients were diagnosed with neuroendocrine tumor (NET) (24.40%) or neuroendocrine carcinoma (NEC) (7.14%). The estimated mean survival was 8.94 ± 0.28 years (95% CI: 8.40-9.48). Male gender, young age, small tumor size and NET tumor type were favorable prognostic factors.

**Conclusion:**

Chinese GEP-NENs patients present characteristics that are similar to American and European patients. However, there is an urgent need to establish a national database for understanding the clinical and epidemiological features of GEP-NENs in China.

## Background

Neuroendocrine neoplasms (NENs) are epithelial tumors with a predominant neuroendocrine differentiation, and they can develop in most organs. This fairly rare neoplasms displays a large spectrum of clinical presentations [1]. According to the Surveillance, Epidemiology and End results (SEER) database, more than half of all NENs are gastroenteropancreatic NENs (GEP-NENs) (61%), with the highest frequency being observed in the rectum (17.7% of NENs), small intestine (17.3% of NENs) and colon (10.1% of NENs), followed by the pancreas (7.0%), stomach (6.0%), and appendix (3.1%) [2]. The annual incidence of GEP-NENs is about 3.65-4.7 cases per 100,000 people in the United States (USA) [2,3]. African Americans show a higher incidence than Caucasians (6.46 vs. 4.60 per 100,000) [2]. The incidence is also slightly higher in men compared with women (4.97 vs. 4.49 per 100,000) [2]. Similar rates were reported in Sweden, Norway, Spain and England [4-7].

None of the published nomenclatures and classifications of NENs present a unified classification that is accepted by clinicians and pathologists [8,9]. In 2010, the WHO presented a new classification of NENs, in which the term NENs describes all tumors with a neuroendocrine differentiation. GEP-NENs can be subdivided into two groups: the well-differentiated neuroendocrine tumors (NETs) and the poorly-differentiated neuroendocrine carcinomas (NECs) [10,11]. Furthermore, NETs and NECs are graded into three types, grades 1 (G1), 2 (G2) and 3 (G3), according to different definitions of proliferation using the mitotic count and/or the Ki-67 index [11]. In general, both G1 and G2 NENs are considered as NETs, and G3 NENs are considered as NECs [11].

Many studies reported the epidemiology, diagnosis, pathology and management of GEP-NENs in the American and the European populations [2,8,12-14], but there is a lack of data in Asian populations, especially in China. Therefore, the objective of the present study was to perform an epidemiological study of GEP-NENs in a Chinese population. The present study might provide new clues about the development and the management of these rare tumors.

## Methods

### Patients

We performed a retrospective analysis of all patients diagnosed with GEP-NENs according to the WHO 2000 classification [15,16] between January 2003 and December 2012 at the First and Second Affiliated Hospitals of Dalian Medical University.

All patients included in the present study had to have received a pathological diagnosis of GEP-NENs, and the original pathology report had to be available. Patients were excluded if they had received a diagnosis of primary NENs of any other site, or if the primary NEN site was unknown. In addition, patients with incomplete records (clinical and pathological), who were lost to follow-up, or who refused to participate in our study were also excluded. The ethics committee of the First Affiliated Hospital of Dalian Medical University approved the study protocol (LCKY 2012–32).

### Data collection

At the Dalian Medical University, all cancer cases are prospectively collected into a database. Therefore, the following variables were collected and analyzed: clinical characteristics (gender, age, symptoms, signs), diagnostic procedures (imagery, pathology), tumor characteristics (primary site, size, stage, grading, World Health Organization (WHO) 2010 classification, WHO 2000 classification), treatments (surgery, hepatic artery interventional chemotherapy, pharmacotherapy), and follow-up (date of diagnosis, date of death and cause of death).

Cancer staging was performed using the usual tumor node metastasis (TNM) approach according to the anatomical sites of the tumors [11,17]. Grading was based on morphological criteria and tumor proliferative activity. Tumors with a Ki-67 index of ≤ 2% were classified as G1, 3-20% as G2, and > 20% as G3. Similarly, tumors with a mitotic rate of < 2 per 10 high power fields (HPF) were classified as G1, 2-20/10 HPF as G2, and > 20/10 HPF as G3. GEP-NENs were further classified as NET (G1 and G2), or NEC (G3), according to the 2010 WHO classification [10,11].

We performed the final follow-up by telephone, mail or outpatient department visit in December 2012.

### Statistical analysis

All statistical analyses were performed using SPSS 20.0 for Windows (IBM Corporation. Armonk, NY, USA). We tested continuous variables for normal distribution. Normally distributed continuous variables are expressed as mean and standard deviation, and were analyzed using independent samples *t*-tests. Categorical variables are expressed as frequencies and proportions, and were analyzed using the chi-square test or Fisher’s exact test, as appropriate. We used the Kaplan-Meier method for survival analysis, log-rank tests were used for comparision among groups and post hoc analysis for pairwise comparisons between groups. We performed Cox proportional hazards model to identify independent factors associated with prognosis. The level of significance was set at *P* < 0.05.

## Results

### Patients’ clinical characteristics

One hundred-sixty-eight patients were included in the present study. All were Han Chinese and Dalian natives; 102 (61.00%) patients were male, 66 (39.00%) female, and the male-to-female ratio was 1.5:1. Mean age was 51.83 ± 14.03. The most frequent initial symptoms and signs were abdominal or back pain (n = 49, 29.17%), followed by gastrointestinal bleeding (n = 43, 25.60%), dyspepsia (n = 24, 14.29%), and diarrhea (n = 21, 12.50%) (Table 1). Eight (4.76%) cases were incidental findings during routine health examinations; these patients were asymptomatic. Seven (4.16%) patients received a diagnosis of functional tumors: all of these were insulinomas, and the patients were hypoglycemic.

**Table 1:** Patients’ characteristics

	**Male**	**Female**	**Total**	***P*** -**value**
**N (%)**	102 (61.00)	66 (39.00)	168 (100.00)	
**Age (years)**	52.91 ± 13.65	50.15 ± 14.56	51.83 ± 14.03	0.06
**Primary tumor site**				
Gastrointestinal tract	93 (55.36)	52 (30.95)	145 (86.31)	0.02*
Stomach	12 (7.14)	4 (2.38)	16 (9.52)	0.22
Duodenum	6 (3.57)	3 (1.79)	9 (5.36)	1.00
Jejunum	1 (0.60)	0(0.0)	1 (0.60)	1.00
Ileum	3 (1.79)	1 (0.60)	4 (2.38)	1.00
Colon	6 (3.57)	2 (1.19)	8 (4.76)	0.48
Appendix	4 (2.38)	4 (2.38)	8 (4.76)	0.71
Rectum	61 (36.31)	38 (22.62)	99 (58.93)	0.74
Pancreas	9 (5.36)	14 (8.33)	23 (13.69)	0.02*
**Clinical Symptoms**				
Abdominal or back pain	27 (16.07)	22 (13.10)	49 (29.17)	0.33
Gastrointestinal bleeding^a^	28 (16.67)	15 (8.93)	43 (25.60)	0.49
Dyspepsia^b^	16 (9.52)	8 (4.76)	24 (14.29)	0.52
Diarrhea	18 (10.71)	3 (1.79)	21 (12.50)	0.12
Tenesmus	13 (7.74)	6 (3.57)	19 (11.31)	0.47
Appetite loss	11 (6.55)	7 (4.17)	18 (10.71)	0.97
Constipation	3 (1.79)	5 (2.98)	8 (4.76)	0.27
Hypoglycemia^c^	5 (2.98)	2 (1.19)	7 (4.17)	0.71
Weight loss	4 (2.38)	1 (0.60)	5 (2.98)	0.65
Asthenia	1 (0.60)	1 (0.60)	2 (1.19)	1.00
Dysphagia	1 (0.60)	0(0)	1 (0.60)	1.00
**Main Signs**				
Abdominal mass	2 (1.19)	2 (1.19)	4 (2.38)	0.65
Jaundice^d^	1 (0.60)	1 (0.60)	2 (1.19)	1.00
Rash	1 (0.60)	2 (1.19)	3 (1.79)	0.56

^a^fecal occult blood, bloody stool, hematemesis.

^b^fullness, bloating, belching, nausea, vomiting.

^c^tremors, cold sweats, palpitations, hunger, irritability, headache, dizziness, blurred vision, disorientation, coma or seizures.

^d^skin or sclera.

### Diagnostic procedures

The following procedures were performed at least once during the diagnosis and management of these tumors: endoscopy (n = 133, 79.17%), ultrasound (n = 82, 48.81%), computed tomography (CT) scan (n = 96, 57.14%), and magnetic resonance imaging (MRI) (n = 17, 10.12%). The highest positive rate was 97.74% (130/133) for endoscopy, followed by endoscopic ultrasound (90.00%), MRI (70.59%), ultrasound (58.54%), and CT (54.17%). Positron emission computed tomography imaging using (^18^ F)-fluoro-deoxy-glucose as tracer (^18^ F-FDG-PET) was performed in four patients, and detected the lesion in three of them, showing a detection rate of 75.00%. Chromogranin A, synaptophysin and neuronspecific enolase (NSE) are general neuroendocrine markers [18], and were positive in 72.62%, 76.19%, 32.74% of patients, respectively (Table 2).

**Table 2:** Diagnostic procedures

	**Cases tested, N (%)**	**Positive, N (%)**
**Imaging diagnosis**		
Endoscopy	133 (79.17)	130 (97.74)
Gastroscopy	26 (15.48)	26 (100.00)
Small intestinal endoscopy	11 (6.55)	8 (72.73)
Proctoscopy	96 (57.14)	96 (100.00)
Ultrasound	82 (48.81)	48 (58.54)
Endoscopic ultrasound	20 (11.90)	18 (90.00)
CT	96 (57.14)	52 (54.17)
MRI	17 (10.12)	12 (70.59)
PET-CT	4 (2.38)	3 (75.00)
**Immunohistochemistry**		
Chromogranin A	168 (100)	122 (72.62)
Synaptophysin	168 (100)	128 (76.19)
NSE	168 (100)	55 (32.74)

CT, computed tomography scan; MRI, magnetic resonance imaging; PET-CT, positron emission computed tomography; NSE, neuron-specific enolase.

### Tumors’ characteristics

As listed in Table 1, the rectum (n = 99, 58.93%) was the most frequent primary site, followed by the pancreas (n = 23, 13.69%), stomach (n = 16, 9.52%), duodenum (n = 9, 5.36%), colon (n = 8, 4.76%), appendix (n = 8, 4.76%), ileum (n = 4, 2.38%), and jejunum (n = 1, 0.60%). There was gender difference in primary tumor site (Table 1).

According to the pathology reports of the 168 tumors, 23 specimens were too small to be properly described (tumor size or extension). The mean size (longest diameter) of the remaining 145 tumors was 2.4 ± 2.3 cm.

At diagnosis, tumor spread was local in 64.29% (n = 108) of patients, loco-regional in 14.29% (n = 24), and metastatic in 8.33% (n = 14) (Table 3). The most common site of distant metastases was the liver (11/14, 78.57%), followed by the peritoneum (n = 2), and bones (n = 1). Two patients presented metastatic tumors in the liver accompanied with brain (n = 1) or ovary (n = 1) metastases. According to the 2000 WHO classification, 7.14% of GEP-NENs (n = 12) were classified as well-differentiated endocrine tumors, 4.17% (n = 7) were classified as well-differentiated endocrine carcinomas, and 7.74% (n = 13) were classified as poorly differentiated endocrine carcinomas. Mitotic rates were missing in all pathology reports. Most reports (n = 115) did not present morphological criteria and the Ki-67 index. According to the available Ki-67 indexes, 20.23% of tumors were G1, 4.17% were G2, and 7.14% were G3. The most common tumor type was NET (n = 41), followed by NEC (n = 12) (Table 3).

**Table 3:** Tumors’ characteristics

	**Stomach**	**Duodenum**	**Jejunum**	**Ileum**	**Colon**	**Appendix**	**Rectum**	**Pancreas**	**Total**
	**n = 16**	**n = 9**	**n = 1**	**n = 4**	**n = 8**	**n = 8**	**n = 99**	**n = 23**	**N = 168 (%)**
**Size**									
<1 cm	2	0	0	0	1	0	39	2	44 (26.19)
1-2 cm	1	4	0	2	1	3	31	8	50 (29.76)
>2 cm	9	5	1	2	5	1	15	13	51 (30.36)
Unclear	4	0	0	0	1	4	14	0	23 (13.69)
**Stage**									
T_1_N_0_M_0_	4	0	0	0	1	2	43	5	55 (32.74)
T_2_N_0_M_0_	1	3	0	0	0	1	17	5	27 (16.07)
T_3_N_0_M_0_	2	1	0	1	2	4	2	7	19 (11.31)
T_4_N_0_M_0_	1	2	0	0	0	0	0	4	7 (4.17)
T_any_N_1_M_0_	5	1	1	3	3	0	10	1	24 (14.29)
T_any_N_any_M_1_	2	2	0	0	2	1	6	1	14 (7.74)
Unclear	1	0	0	0	0	0	21	0	22 (13.69)
**WHO 2010**									
NET/G1	0	2	0	0	1	0	26	5	34 (20.23)
NET/G2	0	1	0	0	1	0	3	2	7 (4.17)
NEC/G3	1	0	0	0	2	0	4	5	12 (7.14)
Unclear	15	6	1	4	4	8	66	11	115 (68.45)
**WHO 2000**									
WDET	0	2	1	0	0	0	5	4	12 (7.14)
WDEC	0	2	0	0	0	0	5	0	7 (4.17)
PDEC	1	2	0	0	0	0	4	6	13 (7.74)
Unclear	15	3	0	4	8	8	85	13	136 (80.95)

TNM: tumor-node-metastasis approach [11]; NET, neuroendocrine tumor; NEC, neuroendocrine carcinoma; G1, grade 1; G2, grade 2; G3, grade 3; WDET, well differentiated endocrine tumour; WDEC, well differentiated endocrine carcinoma; PDEC, poorly differentiated endocrine carcinoma.

### Treatment

Table 4 presents the treatment modalities: 86.90% of patients underwent surgery. The surgical approach in each patient was the most optimal one, tailored to each patient’s disease. Sixteen patients underwent conversion to radical resection after endoscopic resection. Seven patients had postoperative complications (intestinal fistula, seroperitoneum, anastomotic stricture, intestinal obstruction, incision fat necrosis, and anastomotic fistula), and five patients had to be reoperated for their complications.

**Table 4:** Treatment modalities

	**Male**	**Female**	**Total**	***P*- value**
	**N = 102**	**N = 66**	**N = 168**	
**Surgery**	89 (52.98)	57 (33.93)	146(86.90)	0.87
Laparotomy	69 (41.07)	36 (21.43)	105 (62.50)	0.09
Laparoscopic	3 (1.79)	1 (0.60)	4 (2.38)	1.00
Endoscopic	17 (10.12)	20 (11.90)	37 (22.02)	0.04
**TACE**	2 (1.19)	1 (0.60)	3 (1.79)	1.00
**Pharmacotherapy**				
Chemotherapy	14 (8.33)	4 (2.38)	18 (10.71)	0.12
Octreotide	12 (7.14)	12 (7.14)	24 (14.29)	0.25

TACE, transcatheter arterial chemoembolization.

Only 1.79% (n = 3) of patients underwent hepatic transcatheter arterial chemoembolization (TACE) to treat liver metastases. Chemotherapy was the only intervention treatment in five patients with inoperable tumors, and 13 patients underwent chemotherapy as postoperative adjuvant therapy (Table 4). The most commonly used regimen was FOLFOX4 (oxaliplatin, leucovorin and 5-fluorouracil, n = 7), followed by oxaliplatin and capecitabine (n = 5), oxaliplatin and fluoropyrimidine (n = 1), paclitaxel and carboplatin (n = 1), docetaxel and gemcitabine (n = 1), cisplatin and 5-fluorouracil (n = 1), streptozotocin and 5-fluorouracil (n = 1), and taxane and platinum (n = 1). Twenty-four patients (14.29%) received octreotide, a somatostatin analogue, as a biological therapy combined with surgery or chemotherapy. Except for endoscopic therapy, the treatment modality distribution showed no difference in gender for any other therapies.

### Follow-up

**Figure 1:**
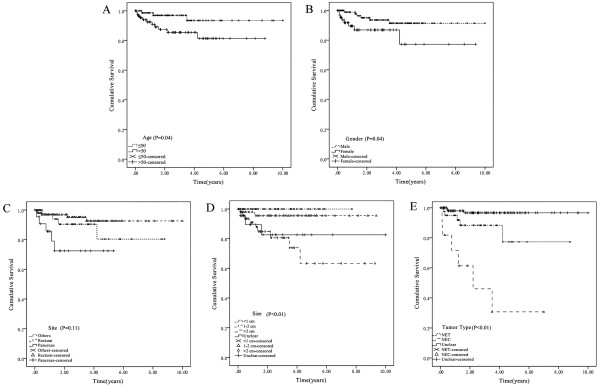
**Kaplan-Meier analyses of different prognostic factors involved in GET-NENs prognosis. A)** Overall survival by age; **B)** Overall survival by gender; **C)** Overall survival by site; **D)** Overall survival by size; **E)** Overall survival by tumor type.

The median follow-up was 2.67 years (range: 0.01-10.00 years). Because of the short follow-up period, the GEP-NENs’ median survival time was not attained during the study. At the last follow-up, 14 patients had died from their GEP-NENs, and 16 patients had died from accidents or other diseases (cerebral thrombosis, lung cancer, myocardial infarction, etc.). The estimated mean overall survival was 8.94 ± 0.28 years (95% confidence interval (CI): 8.40-9.48). We analyzed potential independent survival factors, such as age, gender, primary tumor site, tumor size, and tumor type (NET or NEC). As shown in Table 5, survival was significantly better in young patients, male patients, patients with small tumor and the NET subtype (Figure 1). Multivariate analysis confirmed that age and tumor type were the only independent prognostic factors for overall survival (Table 6).

**Table 5:** Overall survival

	**Number**	**Mean survival times (years)**	**95% CI**	***χ***^***2***^	***P-*** **value**
All	168	8.94 ± 0.28	8.40-9.48		
**Age**				4.33	0.04*
≤ 50	76	9.50 ± 0.29	8.92-10.07		
> 50	92	7.46 ± 0.38	6.73-8.20		
**Gender**				4.25	0.04*
Male	102	9.31 ± 0.27	8.78-9.84		
Female	66	7.71 ± 0.61	6.52-8.91		
**Site**				4.40	0.11
Rectum	99	9.40 ± 0.27	8.88-9.92		
Pancreas	23	4.09 ± 0.48	3.14-5.03		
Others^a^	46	7.59 ± 0.57	6.47-8.72		
Size				18.485	<0.01*
< 1 cm	44	9.84 ± 0.16	9.53-10.15		<0.01^c^
1-2 cm	50	8.88 ± 0.28	8.34-9.42		0.01^c^
> 2 cm	51	5.06 ± 0.51	4.07-6.06		
Unclear^b^	23	8.50 ± 0.39	7.74-9.26		0.01^c^
**Tumor type**				58.840	<0.01*
NET	41	7.35 ± 0.63	6.13-8.58		<0.01^c^
NEC	12	2.80 ± 0.87	1.09-4.50		
Unclear	115	9.80 ± 0.14	9.53-10.08		

^a^others represent stomach, duodenum, jejunum, ileum, colon, appendix.

^b^specimens too small to be properly described tumor size.

^c^log-rank test (pairwise over strata) according to size and tumor type; the *P*-values of tumor size 1 cm vs. > 2 cm, 1–2 cm vs. > 2 cm, Unclear vs. > 2 cm and NET vs. NEC were < 0.05.

*P <0.05.

NET, neuroendocrine tumor; NEC, neuroendocrine carcinoma.

**Table 6:** Multivariate Cox proportional hazards model

**Variable**	**Hazard ratio**	**95% CI**	***P-*** **value**
Age	2.01	1.14-3.56	0.02*
Site	2.94	0.82-10.53	0.10
Size	3.55	0.82-15.47	0.09
Tumor type	2.10	1.02-4.31	0.04*

*P <0.05.

## Discussion

The aim of this retrospective study was to assess the clinical, pathological and prognosis characteristics of GEP-NENs in China. The rectum, pancreas, stomach and duodenum were the most frequent primary sites. The majority of patients presented non-functional tumors with non-specific symptoms such as abdominal or back pain and gastrointestinal bleeding. Based on the 2010 WHO classification, most patients suffered from NET. The estimated mean survival was relatively short, with 8.94 ± 0.28 years (95% CI: 8.40-9.48). Male gender, young age, small tumor and NET tumor type were favorable prognostic factors.

NENs may develop anywhere in the body, but most of them do in the gastrointestinal tract [1]. A large-scale analysis of GEP-NENs (n = 29,664) from the SEER database revealed that the highest GEP-NENs frequency was in the rectum, followed by the small intestine, colon, pancreas, stomach, and appendix, and that the incidence increased with years at all primary sites, especially in the rectum and small intestine [3]. Similarly, we observed that the rectum was the most frequent site of GEP-NENs, followed by the pancreas, and stomach; however, the small intestine only accounted for a small proportion of our cases. Nevertheless, primary tumor site distribution in the present study was similar to that of the Korean and Japanese populations [13,19], suggesting that the distribution of GEP-NENs’ primary sites may be different between the Asian and the American populations. However, these observations might not reflect the true situation. Indeed, guidelines recommend that patients over 50 years undergo colonoscopy when they receive health check at our institutions, which should increase the early diagnosis rate of the disease. Meanwhile, with diagnostic improvements, more and more patients received a diagnosis of appendix NEN because of an incidental finding of the surgery for an acute appendicitis. All of these might create a false impression that the incidence of GEP-NENs is increasing.

NENs can be classified into functional or nonfunctional tumors according to the symptoms associated with peptides and hormones overproduction [20]. The present study showed that most GEP-NENs in the Chinese population were nonfunctional tumors. Carcinoid syndrome, Zollinger-Ellison syndrome, Whipple triad, Verner-Morrison syndrome and glucagonoma syndrome are typical symptoms of functional NENs [21], but no patient showed these symptoms in the present study. This might be due to two reasons: 1) clinicians may not pay enough attention to these symptoms; or 2) NENs were diagnosed at the early stages, before being symptomatic.

The lack of a uniform nomenclature and classification system of GEP-NENs prevent the physicians from diagnosing and treating these tumors. The WHO updated its classification in 2010 and accepted the term “GEP-NENs” [22]. We reviewed the diagnosis process in our participating institutions. Unfortunately, but as expected, it was not standardized. The “gold standard” in our participating institutions was the use of general neuroendocrine markers, such as chromogranin A, synaptophysin or NSE [23]. We reviewed the pathology reports of our patients according to the new WHO 2010 diagnosis and classification criteria, and we observed that 24.40% patients suffered from NET (n = 41) and 7.14% from NEC (n = 12). Fourteen patients presented metastases at diagnosis. The total metastasis rate was 8.33%, which was lower than what was previously reported [24]. The reasons may be responsible for regional difference. Unlike other solid tumors, there is a wide array of therapeutic options, such as surgery, interventional radiology, systemic therapy, somatostatin analogues, interferon, peptide-receptor radionuclide therapy, chemotherapy, and targeted agents (sunitinib, everolimus, bevacizumab) to palliate symptoms and extend survival in patients with GEP-NENs [25]. As for other tumors, surgery is an essential treatment in many GEP-NENs and is usually the only way to cure patients. The extent of surgical resection depends on the extent of the disease (local, regional or distant metastases). Cytoreductive surgery is recommended for palliation and to increase survival for patients with advanced disease [26]. In the present study, most patients underwent surgery, and only two patients who underwent surgery died during follow-up.

Chemotherapy is the first treatment option for inoperable or metastatic GEP-NENs. The cisplatin and etoposide combination is the most widely used chemotherapy regimen for GEP-NENs [27]. In our cohort, 18 patients received chemotherapy. The most frequently used chemotherapy regimen was the oxaliplatin, leucovorin and 5-fluorouracil combination.

More and more studies report that biological and targeted therapies show great promises against NENs. Somatostatin analogues can reduce the hormone-related symptoms, and they are an effective therapeutic option for functional neuroendocrine tumors [28]. Sunitinib and everolimus may be used for patients with inoperable locally advanced or metastatic, progressive, well differentiated pancreatic NENs [2,29,30]. In the present study, 24 patients underwent somatostatin analogues treatment, seven of them suffering from functional tumors (insulinoma).

The present study suffered from some limitations. The population’s characteristics (age and gender) presented in our study are similar to those reported in other studies of Asian populations [13,19,24,31,32]. However, they are different from those previously published using the SEER database or European populations [12,14,33]. Selection biases among races, populations and hospitals may be responsible for these differences. Second, the follow-up of the present study did not reach the median survival time previously reported. Indeed, our mean survival was 8.94 ± 0.28 years, and was shorter than the 9.5 years reported by other investigators [24]. This is due to a short follow-up time and to the slow growing rate of the disease. Third, the small sample size might have been responsible for some bias in the multivariate and prognosis analyses. Fourth, the retrospective nature of the study prevented us from obtaining some information, such as the Ki-67 index. In addition, our follow-up system only contains data about the vital status (alive or not), and not about progression, preventing us to determine the progression-free survival. Finally, tumors in the rectum are more easily found at their early stages by endoscopic examination, while tumors at other sites (e.g. in the pancreas) necessitate examinations that are not routinely performed, which might affect the incidence.

## Conclusion

In our study, non-specific clinical symptoms were the most common symptoms in patients with GEP-NENs. Diagnosis was mainly based on clinical manifestation, endoscopy and imagery, as well as on pathology. However, the recommended morphological criteria and proliferative activity of the tumor were not commonly used in pathological diagnosis. Surgery was the most common intervention. Male gender, young age (≤ 50 years), tumor size (< 2 cm) and NET diagnosis might be favorable prognostic factors. A national database of GEP-NENs should be established for studying the clinical and epidemiological features of these tumors, and to help physicians taking better clinical decisions.

### Consent

Written informed consents were obtained from the patient for the publication of this report and any accompanying images.

## Competing interests

All authors declare that they have no competing interests.

## Authors’ contribution

XZ: conception and design, acquisition of data, writing the manuscript. LM: analysis and interpretation of data. HB: acquisition of data. JZ: acquisition of data, analysis and interpretation of data. ZW: acquisition of data. PG: revising the manuscript. All authors read and approved the final manuscript.

## Acknowledgements

This study was funded by the Specialized Research Fund for the Doctoral Program of Higher Education (No. 20122105110009) and the China National Natural Science Foundation (No.81200989). The funders had no role in study design, data collection and analysis, decision to publish, or preparation of the manuscript.

## Pre-publication history

The pre-publication history for this paper can be accessed here:

http://www.biomedcentral.com/1472-6823/14/54/prepub
